# Transperineal drainage of prostate abscesses: A minimally invasive, low‐risk management strategy that yields satisfactory results

**DOI:** 10.1002/bco2.310

**Published:** 2023-11-27

**Authors:** David Scholtz, Ali Hooshyari, Lodewikus Petrus Vermeulen, Flavio Vasconcelos Ordones

**Affiliations:** ^1^ Urology Department Tauranga Public Hospital Tauranga New Zealand; ^2^ Surgery Department University of Auckland Auckland New Zealand; ^3^ Botucatu School of Medicine São Paulo State University – UNESP São Paulo Brazil

**Keywords:** infection, prostate abscess, transperineal, transperineal drainage, TRUS

## Abstract

**Objectives:**

In this narrative review, we aim to present two cases of transperineal drainage of prostate abscesses with a good clinical outcome. Furthermore, we reviewed the literature on this treatment approach and aim to propose a minimally invasive protocol for managing this rare condition.

**Patients and Methods:**

Our patients are 33‐ and 61‐year‐old males who both underwent uncomplicated transperineal drainage of prostate abscess with the use of a Precision Point device with rapid clinical improvement and complete resolution of the abscess within the follow‐up period. We used PubMed to conduct a literature search and included and evaluated 16 relevant case reports and case series in which the authors utilized transperineal drainage techniques for prostatic abscesses.

**Results:**

Our first patient was young and very unwell with sepsis and a pulmonary embolism. He had a complex abscess extending through the prostate to the left pelvic side wall. Trans‐gluteal drainage of the pelvic side‐wall collection was required in addition to transperineal drainage of the prostate abscess. After drainage and a prolonged course of antibiotics, he achieved resolution of the abscess by 7 weeks with ejaculatory function intact. Our second patient who was very keen on the preservation of ejaculatory function had multiple small abscesses and underwent transperineal drainage. He had significant interval improvement of his abscess burden at the 4‐week follow‐up and complete resolution at the 6‐month follow‐up. The total number of cases in the literature on our review is 22, with considerable variability in how the authors managed the prostate abscesses that underwent transperineal drainage, including variability in their follow‐up time frame, choice of imaging modality, duration of antibiotic treatment, drain placement, and use of irrigation solutions (including antibiotics) into the abscess cavity. Furthermore, the sizes of the prostate abscesses were not consistently reported. Given the small sample size and variability in management from different authors, it was not possible to draw any statistical analysis.

**Conclusion:**

Transperineal prostate abscess drainage combined with prolonged antibiotic therapy provides a less invasive alternative to treating prostate abscesses for those who which to preserve ejaculatory function and avoid the other adverse events of transurethral de‐roofing. In itself, it can achieve complete resolution of abscess. It provides the benefit of drainage under real‐time imaging; for percutaneous drain placement; prevents urethral injury; retrograde ejaculation; and can be done under local anaesthetic which is preferable for the unstable patient. The utility of the procedure may be limited by the complexity of the abscess or whether it has extended beyond the prostate. The patient should always be informed that further drainage via percutaneous methods or transurethral methods may be necessary if their clinical condition does not improve. We recommend this procedure be offered as an alternative to transurethral methods in younger patients and those who would like to preserve ejaculatory function. Furthermore, we highly encourage a prolonged course of antibiotic therapy and interval follow‐up with clinical review of symptoms and imaging to confirm resolution.

## 1 INTRODUCTION

Prostatic abscesses (PA) are rare, affecting approximately only 0.2–0.5% of all men.^1^ There have not been any recent epidemiological studies on the incidence of PA in the general healthy population; however, there are data to suggest that the incidence of PA peaks between the fifth and sixth decades of life and accounts for approximately 0.5–2.5% of all prostatic diseases.^1^ It can, however, cause significant morbidity and mortality if not treated promptly. PA often occurs as a complication of a urinary tract infection or instrumentation of the urinary tract. Risk factors that predispose patients to PA include diabetes mellitus, chronic renal failure, liver cirrhosis, and other immunodeficiency states.^1^ The most common presenting features include irritative lower urinary tract symptoms, fevers, and perineal pain. The most commonly implicated pathogens include *Escherichia coli*, *Klebsiella pneumonia*, *Pseudomonas aeruginosa*,^1^ and mixed‐growth microbes. Transrectal ultrasound (TRUS) is a widely used and inexpensive modality of confirming the diagnosis of PA; however, computed tomography (CT) and magnetic resonance imaging (MRI) scanning are also useful.^2^ Treatment consists of a prolonged course of usually quinolone‐based antibiotics and drainage. Drainage can be achieved by transurethral resection of the prostate abscess (TURP), transurethral de‐roofing of the abscess, TRUS‐guided trans‐rectal drainage, and TRUS‐guided transperineal drainage (TPD). TPD has not been widely studied, and most data on this method come from small retrospective studies and case reports.^1^, ^2^

Herein, we report two cases of prostate abscesses that underwent TPD. Furthermore, we review the literature on reports of TPD of prostate abscesses. We advocate for TPD due to its excellent efficacy and safety profile.

## 2 METHODS

Two cases will be described. Both were done in the operation theatre with patient under general anaesthetics. Precision Point device, normally used for transperineal prostate biopsy, was utilized, that is, in fact, what differentiate our two cases from the rest of the series or case report published so far. As far as we know, these are the first two cases of a prostatic abscess drained with the use of the Precision Point device published in the literature.

With regard to the review, we used the PubMed database to conduct a literature search. We set the time frame between 1 January 1975 and 1 January 2023. The keywords ‘transperineal drainage’ and ‘prostate abscess’ were entered. A total of 55 results were obtained. Of these, 20 were either case series or case reports in which at least some (if not all) patients had TPD of PA. Of these 20 articles, we were only able to gain access to 16 of them. Three articles that were excluded were in an inaccessible Spanish journal (single case reports each) and the other a case series in Russia (of which 19 patients underwent TPD of PA as per the abstract). Of the 16 articles we used, we identified 22 cases of TPD for PA (see Table 1). Across these 16 reports we did utilize, three were case series with more than one patient in each. The remainder were case reports with single patients. It should be noted that there was significant variability in the number of details in each case report. Some parameters which were not consistently specified by all authors include size of prostate abscess; causative organism; choice of and duration of antimicrobial therapy; post‐discharge follow‐up; and time to follow‐up. Specific parameters that we were interested in include the size of the prostate abscess; the placement of a drain; irrigation of the abscess cavity; the causative organism; duration of antibiotic therapy; time to complete resolution of abscess; and follow‐up interval.

**TABLE 1:** A list of the case reports and case studies in which transperineal drainage of prostate abscesses was performed.^3^, ^4^, ^5^, ^6^, ^7^, ^8^, ^9^, ^10^, ^11^, ^12^, ^13^, ^14^, ^15^, ^16^, ^17^, ^18^

Case	Author(s)	Age	Comorbidities	Presenting complaint
1	Shapiro and Sherer (1975)	59	None	Acute urinary retention
2	Kadmon and Ling (1986)	47	Alcoholic cirrhosis End stage renal failure	Testicular pain and fever
3	Kadmon and Ling (1986)	50	Type II diabetes mellitus End‐stage renal failure on dialysis Previously rejected kidney transplant	Fever
4	Sugao et al. (1986)	54	None	Dysuria, frequency, and fevers
5	Sugao et al. (1986)	55	Cervical spondolytic radiculopathy	Rectal pain, dysuria, and fevers
6	Cytron et al. (1988)	*72*	Type II diabetes mellitus	Dysuria, frequency, fever, and weight loss
7	Cytron et al. (1988)	63	Type II diabetes mellitus Ischaemic heart disease	Dysuria and fever
8	Cytron et al. (1988)	44	Previous urethritis	Frequency, dysuria, and perineal pain
9	Cytron et al. (1988)	44	None	Fever, dysuria, haematuria, and suprapubic pain
10	Cytron et al. (1988)	28	Recurrent urinary tract infections	Fever, urinary retention, and back pain
11	Rørvik and Daehlin (1989)	50	Thyrotoxicosis	Dysuria, frequency, and acute urinary retention
12	Serrano et al. (2006)	38	None	Dysuria, fever, and testicular and perineal pain
13	Machida et al. (2008)	81	Chronic obstructive pulmonary disease	Left groin pain and swelling
14	Faris et al. (2008)	47	None	Dysuria, fever, and tender prostate on exam
15	Mason et al. (2010)	83	None	Abdominal pain with sepsis
16	Galosi et al. (2010)	62	Penile cancer Hypertension Type II diabetes mellitus Ischaemic heart disease	Dysuria
17	Arrabal‐Polo et al. (2011)	84	Hypertension Chronic kidney disease	Nocturia, frequency, perineal pain, and fevers
18	Choudhry et al. (2017)	55	Human immunodeficiency virus infection Type II diabetes mellitus Chronic pancreatitis	Urethral discharge and frequency and poor stream
19	Okumura et al. (2018)	43	Type II diabetes mellitus	Dysuria
20	Hoe et al. (2019)	61	Prostate cancer Radiotherapy to prostate and SpaceOAR insertion Ulcerative colitis	Urethral discharge, anal pain, fever, and weight loss
21	Hajji et al. (2021)	51	Type II diabetes mellitus	Dysuria, frequency, pelvic pain, fever, penile pain, and swelling
22	Yokoyama et al. (2022)	81	Myelodysplastic syndrome	Abdominal pain and fever

*Note*: A total of 22 patients underwent transperineal drainage of their prostate abscesses. Comorbidities and presenting symptoms are also listed.

### 2.1 Technique description

Patients were anaesthetized and placed in the lithotomy position. The perineum was prepped with povidone iodine, and a sterile drape was placed. A BK® Specto Ultrasound with a biplanar probe was placed in the rectum to provide real‐time images of the prostate. The PrecisionPoint® transperineal access system was secured onto the biplanar probe as it would be for transperineal prostate biopsies. The sliding carrier was utilized but the access needle was removed. The needle used for drainage was a 15‐cm‐5fr‐one‐step needle. This needle fits exactly in the sliding carrier and the number of punctures varied according to the number of abscesses visible on ultrasound. A 20‐mL syringe was used to aspirate the abscesses(s) until either the abscess cavity was no longer visible on ultrasound or no further contents were aspirated. The samples were sent to the laboratory for culture and sensitivities.

### 2.2 Cases

#### 2.2.1 Case 1 (length of stay of 10 days)

**FIGURE 1:**
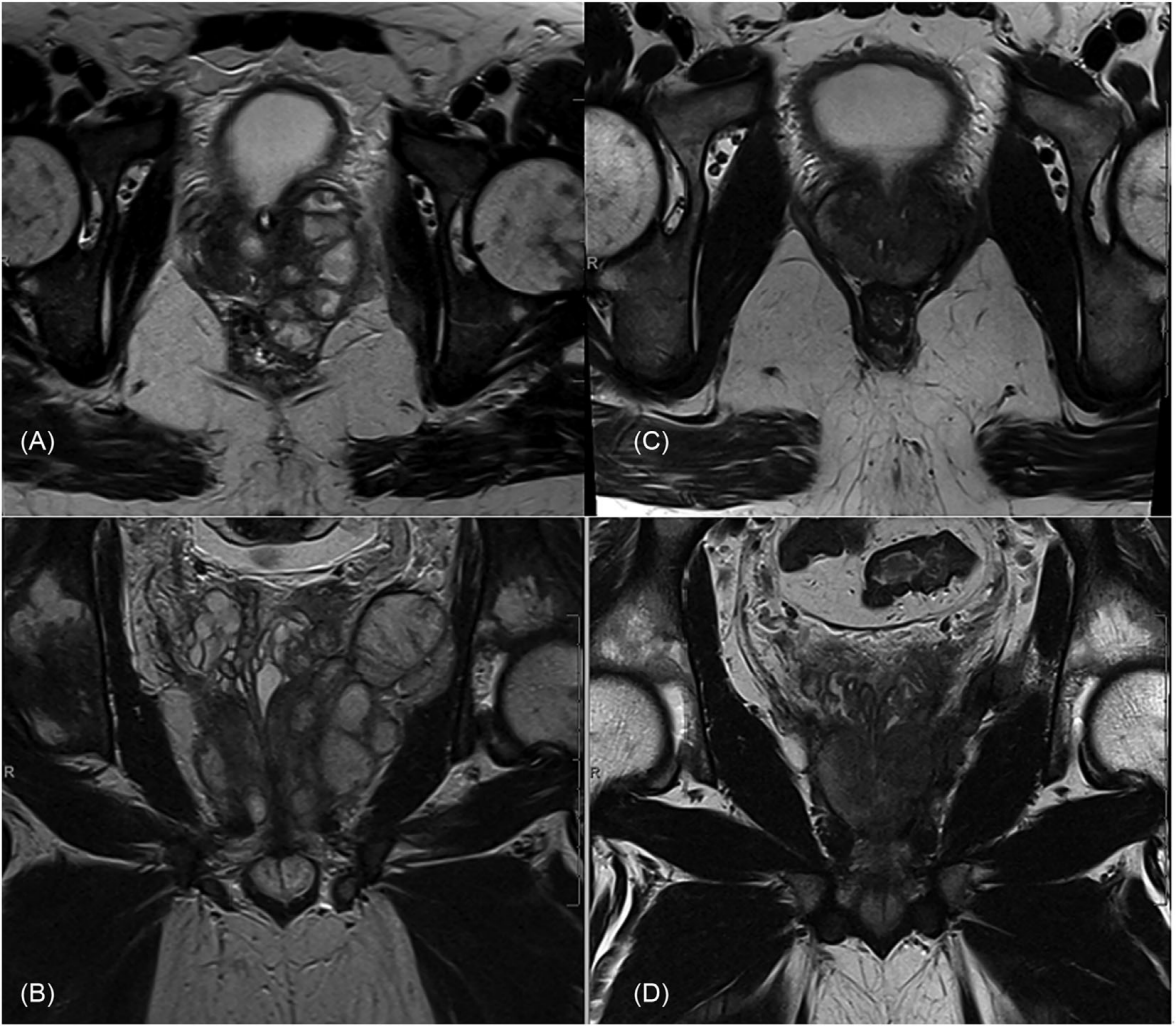
MRI images showing the prostate abscess for Case 1, a 33‐year‐old male. (A) Axial view and (B) coronal view are images obtained at diagnosis. Note how the collection extends into the left pelvic wall. (C) Axial view and (D) coronal view were obtained at 7 weeks post‐drainage and show near complete resolution of the abscess.

A 33‐year‐old male presented with right flank pain, vomiting, fevers, and a 12‐kg weight loss over 4 weeks. He had a urinary tract infection 4 weeks prior and no other past medical history. On examination, he looked unwell, was febrile (38.3°C), and tachycardic (113 bpm), with right flank tenderness. Immediate fluid resuscitation was initiated, and the patient was commenced on intravenous Cefuroxime. Significant initial blood results include the following: Haemoglobin (Hb) 116 g/L; white cell count (WCC) 18.5 × 10^9^ cells; neutrophils 16.8 × 10^9^ cells; C‐reactive protein 324 mg/L; and a blood culture positive for *K. pneumonia*. A urinalysis and sexual health screen were unremarkable. Human immunodeficiency virus serology and COVID‐19 polymerase chain reaction (PCR) testing were negative. A CT scan showed a complex mixed cystic‐solid lesion (5.7 cm × 6 cm × 6.5 cm) involving the prostate and left seminal vesicle, extending supero‐laterally to the left pelvic side wall. An MRI was obtained for surgical planning which showed the abscesses and an estimated prostate size of 3.7 cm × 3.9 cm × 3.6 cm (see Figure 1A,B). He was interested in an ejaculatory sparing intervention. TPD of the abscess was performed under general anaesthetic. Fifteen millilitres of frank pus was drained, and his cultures returned with *K. pneumonia*. Antibiotics were then switched to Ertapenem. He remained tachycardic and febrile in the first 24 h post‐drainage, so an interval CT scan was obtained and demonstrated a right lower lobe pulmonary embolus for which we commenced anticoagulation. This was thought to be a complication of the sepsis rather than the procedure based on the timing of the diagnosis, how unwell the patient was pre‐drainage, and the minimal invasiveness of TPD. The CT also demonstrated a smaller but persistent pelvic side‐wall collection which was drained through a trans‐gluteal approach. After 48 h of no fevers and down‐trending inflammatory markers, he was discharged 10 days after admission with an indwelling catheter and trans‐gluteal drain in situ for a week. A 6‐week course of cotrimoxazole antibiotics was given (in accordance with sensitivities). On a follow‐up MRI scan 7 weeks later, there was near complete resolution of the abscess (see Figure 1C,D).

#### 2.2.2 Case 2 (length of stay of 4 days)

**FIGURE 2:**
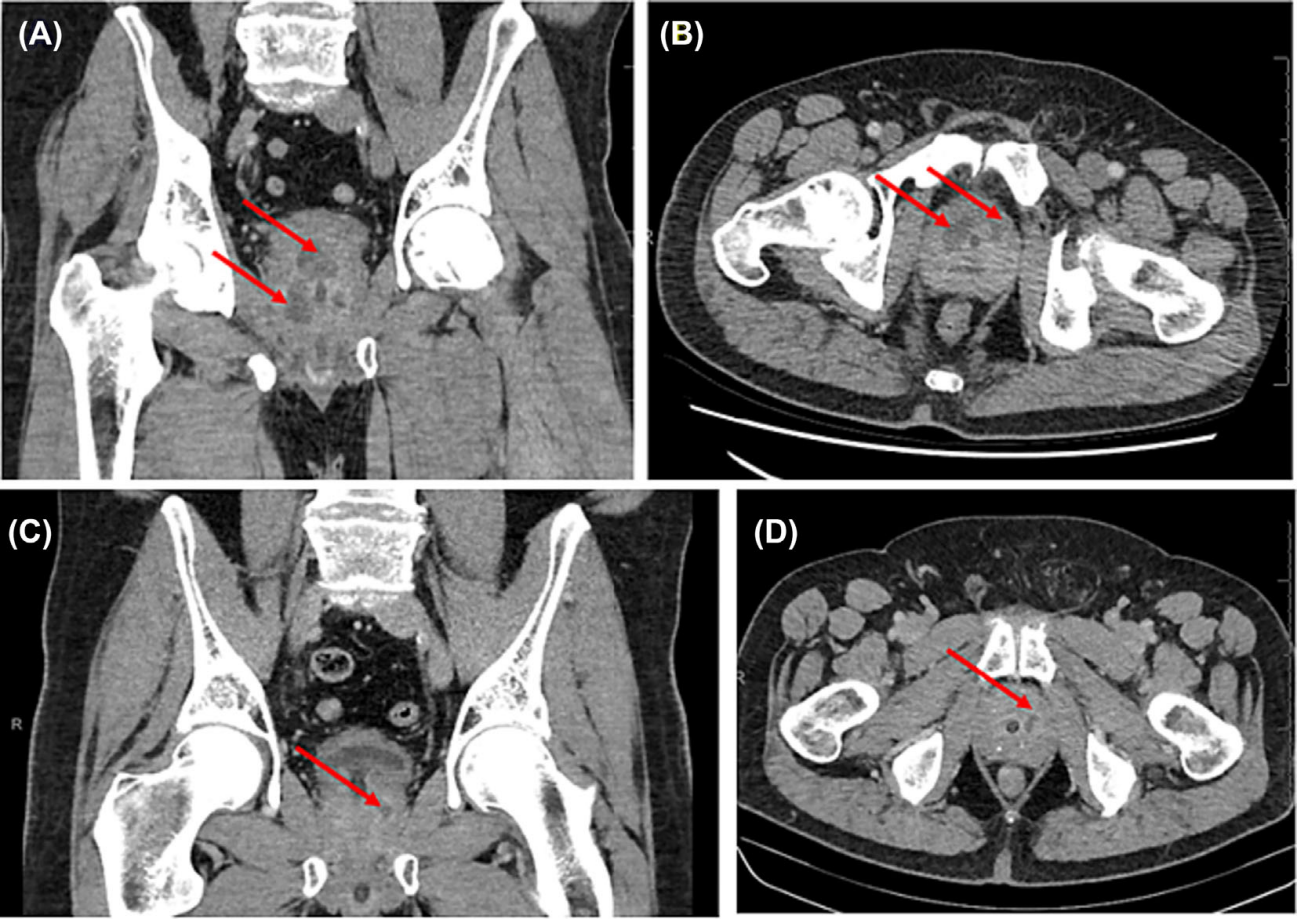
CT images showing the prostate abscess for Case 2, a 61‐year‐old male. (A) Coronal view and (B) axial view show multiple prostate abscesses. (C) Coronal view and (D) axial view were obtained 4 weeks post‐drainage and show a smaller, single 2 cm × 1 cm collection.

A 61‐year‐old male presented with mixed irritative and obstructive lower urinary tract symptoms, fevers, and purulent discharge from the urethra. He had a urethral indwelling urinary catheter in situ for urinary retention. On examination, he looked well, with normal vital signs, slight right renal angle tenderness, and a very tender prostate on digital rectal examination. Significant initial blood results include the following: Hb 127 g/L, WCC 9.2 × 10^9^ cells, neutrophils 7.3 × 10^9^ cells, and C‐reactive protein 150 mg/L. A urinalysis and sexual health screen were unremarkable. HIV serology and COVID‐19 PCR testing were negative. A CT scan showed multiple hypodensities scattered throughout the prostate suspicious for abscesses (see Figure 2A,B). Our team has discussed a transurethral approach; however, the patient wanted to preserve ejaculatory function. Therefore, TPD of the abscess was performed under general anaesthetic, and his cultures returned with extended spectrum beta‐lactamase (ESBL) *E. coli* (sensitive to ciprofloxacin). He stabilized over the next 48 h and was discharged with 2 weeks of ciprofloxacin and an indwelling urinary catheter to remain in for 4 weeks. He had a follow‐up CT at 4 weeks post‐discharge which showed a residual 2 cm × 1 cm focus within the prostate still likely to be recurrent collection for which he continued antibiotics only (see Figure 2C,D). His symptoms had essentially resolved by the 12‐week follow‐up. The patient then went on a holiday overseas so a repeat MRI was done at 6 months post‐drainage which showed no residual collection, however a new PIRADS 4 lesion. He underwent a Transperineal Prostate Biopsy under local anaesthetics, covering whole prostate template and target lesion. His prostate specific antigen (PSA) was fluctuating between 9 and 10 μg/L with a PSA density of 0.11. Final histopathology did not demonstrate any cancer.

## 3 RESULTS

The mean age of the 22 reported cases that underwent TPD in this review is 57 ± 15 years (mean ± standard deviation). In those cases where the authors reported the size of the prostate abscesses (see Table 2), the mean largest dimension is 51.8 mm ± 25.6 mm (mean ± *SD*). Of the 22 patients, 17 (77%) had a drain placed post‐drainage of the abscess, four (18%) did not, and for one (5%) patient, drain placement was not specified by the author. Of those who had drains placed, 13 (76%) had complete resolution of the prostate abscess by discharge date, and four (24%) did not. Of those without drains, three (75%) had complete resolution of the abscess by discharge, and one (25%) did not (see Table 2). Of all those with drains placed, seven patients had some form of irrigation of the abscess cavity through the drain (see Table 2). Cytron et al. had five patients who had irrigation of the abscess cavity with 1% Neomycin at the time of drainage only. Four of their five patients had a documented abscess size of >20 mm; whereas one had a diffusely abnormal prostate with multiple small abscesses. The four patients with single large abscesses had complete resolution of their prostate abscess within 48 h. The patient with multiple abscesses had complete resolution by 3 months. Galosi et al. performed daily combined saline and unspecified antiseptic solution flushes into the abscess cavity through the drain for 17 days total. This patient had complete resolution of their prostate abscess on discharge. Hoe et al. performed daily saline and gentamicin flushes into the abscess cavity of their patient for 4 days until the drain was removed. They too had resolution of the prostate abscess on discharge. Of the seven patients who had some form of irrigation post‐TPD, six had complete resolution of their prostate abscess between 1 and 17 days. Of the 17 cases that reported the isolated pathogen, *E. coli* (59%) was the most common organism followed by *Staphylococcus aureus* (24%), mixed growth (12%), and *K. pneumonia* (5%). The antibiotics and duration of treatment were inconsistently reported and is represented in Table 3. Follow‐up imaging varied between case reports and ranged from 2 weeks to 6 months post‐discharge. For those who were reimaged within 2 months of admission, either complete or near complete resolution of the prostate abscesses was noted. For those who had imaging at 6 months, there was complete resolution. No patient had recurrence or new abscesses during their follow‐up period. In none of the cases were any TPD‐specific complications mentioned.

**TABLE 2:** A list of the prostate abscess sizes that were reported and whether drains were placed and irrigation was used.

Author(s)	Abscess size	Drain in situ (post TPD)	Irrigation of abscess cavity	Time to resolution	Recurrence
Cytron et al. (1988)	22 × 7 mm and 12 × 4 mm (TRUS)	Yes	1% neomycin solution	1 day (confirmed with TRUS)	No
Cytron et al. (1988)	45 mm × 23 mm (TRUS)	Yes	1% neomycin solution	2 days (confirmed with TRUS)	No
Cytron et al. (1988)	27 mm × 17 mm (TRUS)	Yes	1% neomycin solution	2 days (confirmed with TRUS)	No
Cytron et al. (1988)	29 mm × 23 mm (TRUS)	Yes	1% neomycin solution	1 day (confirmed with TRUS)	No
Rovrik and Daehlin et al. (1989)	35 × 25 mm (TRUS)	No	No	1 week (confirmed by TRUS)	No
Mason et al. (2010)	82 mm × 75 mm × 97 mm (CT scan)	Yes (Stamey percutaneous suprapubic Malecot catheter)	No	10 days (confirmed on CT scan)	No
Galosi et al. (2010)	83 mm × 51 mm (TRUS and CT scan)	Yes (8Fr pigtail drain)	Saline and antiseptic solution flush	17 (assumed as drain output ceased and inflammatory markers normalized)	No
Arrabal‐Polo et al. (2011)	25 mm × 15 mm (TRUS)	Yes (10Fr nephrostomy tube)	No	3 days (confirmed on TRUS)	No
Choudhry et al. (2017)	56 mm × 5.6 mm (MRI)	Yes (drain not specified)	No	4 weeks (confirmed by MRI)	No
Hoe et al. (2019)	54 mm × 35 mm 75 mm	Yes (pigtail tube)	No	Significantly reduced size to 26 mm × 27 mm × 15 mm at 2 months (confirmed by CT scan)—no other follow‐up	No
Haji et al. (2021)	61 mm × 60 mm × 76 mm (TRUS and MRI)	Yes (7Fr double lumen central venous catheter)	Saline and gentamicin flush	6 months (TRUS shows complete resolution)	No

*Note*: Also, not the time to resolution for each case and the lack of recurrence of prostate abscesses at follow‐up.^6^, ^7^, ^11^, ^12^, ^13^, ^14^, ^16^, ^17^ Drain types were included.

Abbreviations: TPD, transperineal drainage; TRUS, transrectal ultrasound.

**TABLE 3:** A list of the organisms that were reported, antibiotic choices both during admission and on discharge, and the duration of treatment.^3^, ^4^, ^5^, ^6^, ^7^, ^9^, ^12^, ^13^, ^14^, ^15^, ^16^, ^17^, ^18^

Author(s)	Organism	Inpatient antibiotic(s)	Outpatient antibiotics(s)	Duration of antibiotics on discharge
Shapiro and Sherer (1975)	*Escherichia coli*	Ampicillin and gentamicin	Not specified	Not specified
Kadman and Ling (1986)	*E. coli*	Not specified	Not specified	Not specified
Sugao et al. (1986)	*E. coli*	Not specified	Not specified	Not specified
Sugao et al. (1986)	*Staphylococcus aureus*	Not specified	Not specified	Not specified
Cytron et al. (1988)	*Klebsiella pneumonia*	Cefuroxime, cotrimoxazole, and amikacin	Cotrimoxazole	3 months
Cytron et al. (1988)	*E. coli*	Cotrimoxazole	Cotrimoxazole	3 months
Cytron et al. (1988)	*E. coli*	Cotrimoxazole	Nitrofurantoin	Not specified
Cytron et al. (1988)	*E. coli*	Cefuroxime	Cotrimoxazole	3 months
Cytron et al. (1988)	No growth obtained	Cefuroxime	Cotrimoxazole	3 months
Rorvik and Daehlin (1989)	*E. coli*	Netilmicin and penicillin	Not specified	Not specified
Machida et al. (200)	*E. coli*	Not specified	Not Specified	Not specified
Galosi et al. (2010)	*E. coli*	‘Broad spectrum antibiotics’	Not specified	Not specified
Arrabal‐Polo et al. (2010)	*E. coli*	Meropenem and amoxicillin–clavulanic acid	Amoxicillin–clavulanic acid	Not specified
Choudhry et al. (2017)	*E. coli* and *Proteus mirabilis*	Unspecified carbapenem and amikacin	Ciprofloxacin	6 weeks
Okumur et al. (2018)	*S. aureus*	Tazobactam–piperacillin and clindamycin	Clindamycin	Not specified
Hoe et al. (2019)	*Streptococcus anginosus* and mixed anaerobes	Not specified	Not specified	5 weeks
Hajji et al. (2021)	Methicillin‐resistant *S. aureus*	Cetriaxone, gentamicin, and vancomycin	Cotrimoxazole	Unspecified
Yokoyama et al. (2022)	*S. aureus*	Not specified	Not specified	Not specified

*Note*: In some of the studies, not all the relevant data were reported.

## 4 DISCUSSION

There exist no strict guidelines for the management of prostate abscesses. Smaller abscesses may respond to antibiotic therapy alone, whereas larger abscesses may be treated with prolonged courses of antibiotics only but require closer surveillance.^19^ Larger abscesses generally resolve quicker with more satisfactory clinical outcomes if they are drained. Many authors in the literature suggest that abscesses where the largest diameter is <2 cm (considered small), an initial trial with antibiotic therapy is appropriate and will often lead to complete resolution. Those PA larger than 2 cm seem to make a better recovery if they are drained.^19^, ^20^ The most frequently used antibiotics for the treatment of prostate infections are ciprofloxacin or cotrimoxazole.^1^, ^21^ Both agents show excellent tissue penetration within the prostate and reach prostate fluid. Empirically third generation cephalosporins and gentamicin are often used; however, they have poorer bioavailability in the prostate, particularly gentamicin whose concentration within prostatic tissue is far below the minimum inhibitory concentrations for most of the common pathogens implicated in bacterial prostate infections. Carbapenems have reasonable prostate tissue bioavailability and may be useful for ESBL pathogens implicated in bacterial prostate infections.^22^ It is therefore paramount to establish a diagnosis in a timely manner to avoid delaying effective antibiotic therapy. There is no set duration of treatment; however, most authors suggest for a minimum of 2–4 weeks of antibiotic therapy, and even up to 6 weeks.^2^, ^21^, ^23^ Decisions regarding the treatment method may be based on patient comorbidities and clinical status, both factors that may influence prognosis. Chang et al. conducted a retrospective single centre case–control study on 44 men over 10 years. Nineteen men were successfully treated with antibiotics alone. Five patients had transurethral de‐roofing of the abscess, and 20 had TURP in addition to antibiotics. The decision to opt for surgical intervention were large abscesses (>2.2 cm); large prostates (>48 g); or a white cell count >17. All patients had complete resolution of abscess and symptoms; however, the intervention group generally had a longer hospital stay, possibly related to the severity of disease.^24^

**FIGURE 3:**
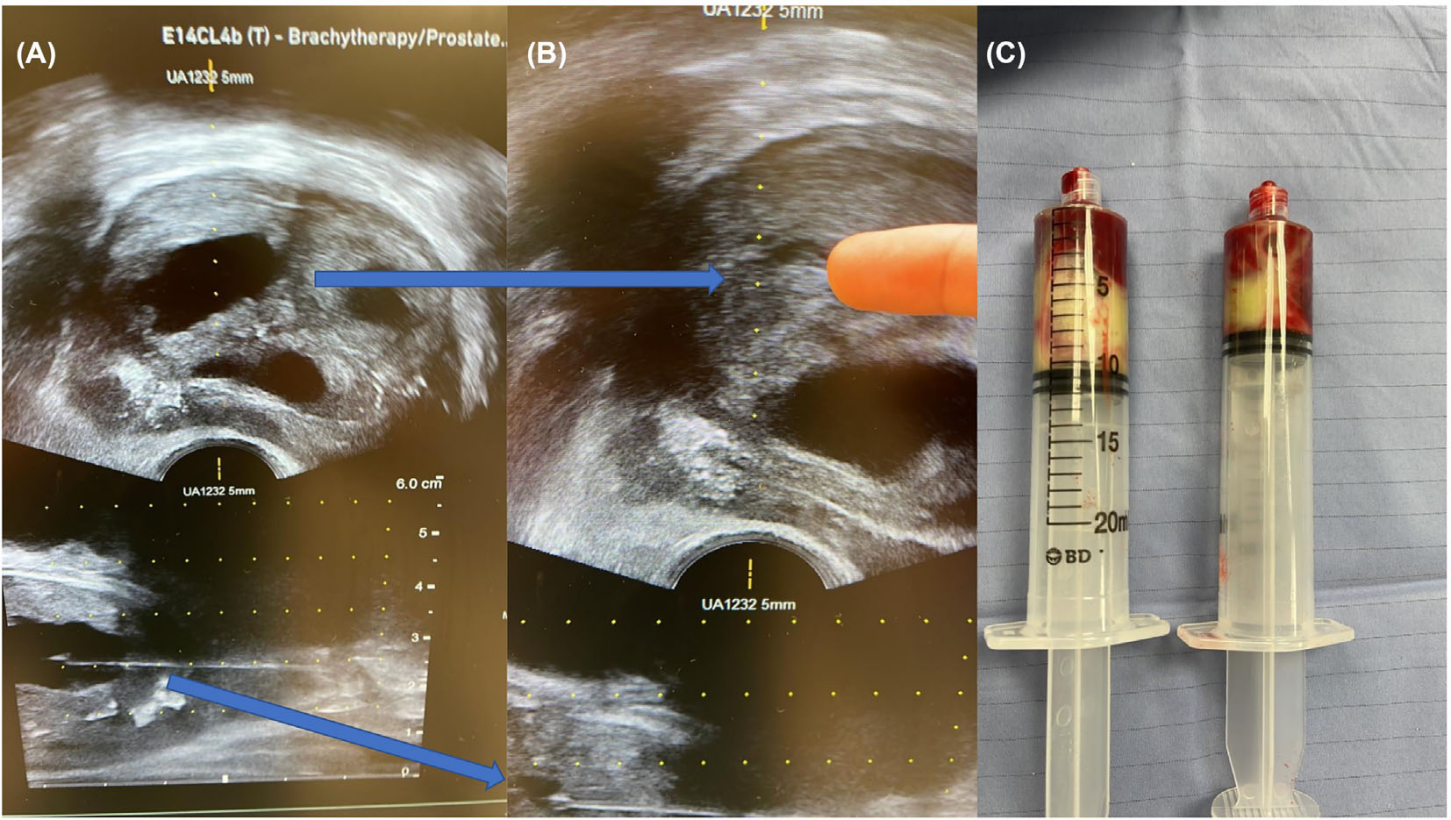
Axial and sagittal views of prostate abscess. (A) The prostate with three “pockets” and the top right one being drained. (B) The previously mentioned “pocket” was emptied. (C) Fifteen millilitres of pus removed after all the abscesses were drained.

The most studied method for treating PA is TURP. TURP is effective with complete resolution in nearly all cases, with the lowest recurrence rate among all drainage techniques.^21^ It is the preferred method in larger prostates (> 80 g) and in instances where less invasive methods have failed.^25^, ^26^ It may be limited by its ability to safely and successfully drain peripheral abscesses.^27^ TURP generally requires either general anaesthesia or spinal anaesthesia. The risks associated with TURP are ejaculatory dysfunction, meatal stricture, urethral stricture, urinary incontinence, and epididymoorchitis.^28^ Trans‐rectal drainage is another method; however, this carries significant risk for sepsis and rectal bleeding.^29^, ^30^ We advocate for a minimally invasive approach through utilizing TRUS‐assisted TPD for multiple reasons. Transperineal instrumentation of the prostate is becoming commonplace in urological practice and is highly recommended over transrectal approaches by the European Association of Urology for prostate biopsy due to higher accuracy and lower infection risk.^31^ As such, the imaging technology is constantly improving, and surgeons are becoming more skilled at the procedure. The transperineal approach provides percutaneous drainage of the PA under real‐time imaging (Figure 3A–C). The biplanar probe utilized for transperineal prostate biopsy provides sagittal and axial views of the entire prostate, which allows the surgeon to target specific areas of the prostate involved in the abscess, thereby sparing healthy tissue. TPD allows the surgeon to place a drain in the abscess cavity. One limitation of TPD may be the patient's inability to tolerate the procedure; however, recent studies of transperineal prostate biopsies in the outpatient setting with advances in peri‐prostatic and perineal blocks have proven that it is tolerable and can be done safely under a local anaesthetic.^32^, ^33^ As such, TPD would be ideal in the septic and unstable patient. Another limitation may be incomplete drainage leading to recurrence and subsequent need for transurethral interventions; however, this appears to be relatively rare and more likely to occur with multiloculate, irregular walled abscesses.^21^, ^23^ TPD avoids placing significant hydrostatic pressure on infected tissue (which may occur during transurethral approaches), thereby avoiding bacterial translocation into the systemic circulation and worsening sepsis.^34^ The severe complication rate of transperineal instrumentation of the prostate is low and this was demonstrated in a study by Pepe and Pennisi where the authors found that out of 8500 men undergoing transperineal prostate biopsy, only 1.5% were readmitted to hospital with fever or acute urinary retention. Not surprisingly, severe adverse effects were more likely in patients where >12 cores were taken and even higher when >18 cores were taken. All other side effects were largely self‐limiting (mainly haematuria and haemospermia).^35^ Drainage of PA is likely to be less invasive compared with prostate biopsy, as the needle is carefully placed into the abscess cavity under TRUS guidance rather than punctures to healthy prostate tissue, therefore injury to healthy tissue can be minimized. TPD also avoids injury to the ejaculatory structures that may lead to ejaculatory dysfunction and is therefore certainly a more favourable option for younger patient who wish to preserve ejaculatory function.

Our first case presented extremely unwell with sepsis and a large abscess burden (5.7 cm × 6 cm × 6.5 cm) which had already extended beyond the prostatic capsule into the left pelvic side wall. He also developed a pulmonary embolus which we believe was provoked by his sepsis rather than the TPD procedure. Patients with severe sepsis have up to a 37% risk of deep venous thromboembolism and up to 3.5% risk of developing pulmonary embolism.^36^ This is thought to be driven by endothelial dysfunction and dysregulation of the various coagulation pathways, favouring a pro‐thrombotic environment.^37^ Post‐operative pulmonary embolism risk correlates directly with the duration of surgery, the invasiveness of surgery, and preoperative risk factors.^38^ Sepsis aside, our patient's baseline characteristics and the fact that he underwent a minimally invasive procedure support a relatively low risk of deep venous thrombosis. Finally, in combination with the aforementioned factors, the fact that he was diagnosed with a pulmonary embolism within 24 h of the procedure also makes a procedure‐related deep vein thrombosis less likely, given that these are usually diagnosed 2–11 days post‐surgery.^39^ TPD of the prostate abscess, trans‐gluteal drainage of the small pelvic side‐wall collection, and a prolonged course of antibiotics achieved complete resolution of the abscess and associated symptoms by 7 weeks, and the patient had preserved ejaculatory function. The extent and severity of his abscess likely account for the long time to resolution; however, resolution was achieved without exposing him to the risks of TURP. Our second case had multiple complex abscesses which had largely resolved by the 4‐week follow‐up, with only a small residual collection (2 cm × 1 cm) remaining after 4 weeks, and complete resolution of symptoms by 12 weeks after a prolonged course of antibiotics. As evident from our two cases and our literature search, the efficacy of TPD of PA is high. These findings are consistent with that reported in a case series by Barozzi et al. where they performed TPD in five patients with abscesses ranging in maximum dimensions from 15 to 40 mm. These patients had rapid resolution of symptoms and normal TRUS findings by Day 15 post‐diagnosis.^40^ A systematic review and meta‐analysis of treatment modalities for PA by Khudur et al. found TPD to be rarely used; however, complete resolution was seen in most cases within 1 month of diagnosis and recurrence is uncommon.^21^ Neither of our two cases had transperineal drains placed or irrigation of the abscess cavity. In our review of the literature, 77% of patients undergoing TPD had a drain placed, 18% did not, and in one case, drain placement was not specified. The resolution of abscess by the date of discharge was 76% and 75% in the group with a drain and group without a drain, respectively. Furthermore, seven patients had some form of irrigation of their abscess cavity, of which six had complete resolution by discharge date (ranging from a hospital stay length from 1 to 17 days). Though these numbers are too small to draw any clinically statistically significant inferences, the trend suggests that there is no major difference in the outcome between drain placement or not. Therefore, the placement of a drain may be at the discretion of the surgeon. Whether irrigation of the abscess cavity provides any benefit is a matter of debate and that certainly requires further investigation. Irrigation of abscesses is not common and in fact is not recommended for superficial abscesses.^41^ We could not identify any studies that assessed the benefit of irrigation of deep abscesses. There is a utility for irrigation peri‐operatively for some intra‐abdominal surgery (such as appendicectomy) in order to reduce the risk of subsequent abscess formation.^42^

## 5 CONCLUSION

TPD appears to be an effective, minimally invasive, ejaculatory sparing approach to treat PA. It provides the benefit of drainage under real‐time imaging for a targeted approach; allows for percutaneous drain placement; avoids injury to the urethra and preserves ejaculatory function; and can be done under local anaesthetic which is preferable for the unstable patient. PA is a rare disease entity, and therefore, the practicality of conducting a trial to compare its efficacy and adverse effect profile to other treatment modalities is limited. Long‐term data are not available in the literature, and our presented cases have less than 1‐year follow‐up. However, with the advances in technology and increased skills of surgeons in transperineal prostate instrumentation for prostate biopsy purposes, we advocate for TPD as at least an option for PA drainage to limit adverse effects for the patient.

## AUTHOR CONTRIBUTIONS

David Scholtz contributed to the data collection, writing, and literature review of this study. Ali Hooshyari contributed to the literature review and data collection. Lodewikus Petrus Vermeulen contributed to the article review and data collection. Flavio Vasconcelos Ordones contributed to the concept, organization, guidance, and article review (senior author).

## CONFLICT OF INTEREST STATEMENT

The authors declare no conflicts of interest.
